# Distributional reaction time properties in the Eriksen task: marked differences or hidden similarities with the Simon task?

**DOI:** 10.3758/s13423-013-0561-6

**Published:** 2013-12-04

**Authors:** Borís Burle, Laure Spieser, Mathieu Servant, Thierry Hasbroucq

**Affiliations:** 1 Aix-Marseille Université & CNRS, Marseille, France; 2 Laboratoire de Neurosciences Cognitives, Aix-Marseille Université, CNRS, Case C 3, Place Victor Hugo, 13331 Marseille, cedex 3, France

**Keywords:** Stimulus–response compatibility, Cognitive control, Automaticity, Electrophysiology, Response time models

## Abstract

In conflict tasks, the irrelevant stimulus attribute needs to be suppressed for the correct response to be produced. In the Simon task, earlier researchers have proposed that this suppression is the reason that, after an initial increase, the interference effect decreases for longer RTs, as reflected by late, negative-going delta plots. This view has been challenged by observations of positive-going delta plots, even for long RTs, in other conflict tasks, despite a similar necessity for suppression. For late negative-going delta plots to be interpreted as reflecting suppression, a necessary, although maybe not sufficient, condition is that similar patterns should be observed for other conflict tasks. We reasoned that a similar suppression could be present, but hidden, in the Eriksen flanker task. By recording and analyzing electromyograms of the muscles involved in response execution, we could compute delta plots separately for trials that elicited a subthreshold incorrect response activation (*partial error*). Late negative-going delta plots were observable on partial-error trials, although they were weaker than for the Simon task, reducing the impact of this inversion on the overall distribution. We further showed that this pattern is modulated by time pressure. Those results indicate that mechanisms leading to negative-going delta plots, similar to those observed in the Simon task, are also at play in the Eriksen task. The link between negative-going delta plots and executive online control is discussed.

When facing complex environments, prioritizing the processing of relevant information is essential to keep adaptive behavior. In the laboratory, such situations are typically studied in so-called “conflict” or “compatibility” tasks, such as the Stroop (1935), the Simon (1969), or the Eriksen flanker (Eriksen & Eriksen, 1974) tasks. In all of those tasks, the stimuli have two dimensions, one to which the participants are instructed to respond to (e.g., the color of the font in the Stroop task), and a second one that, although it is irrelevant for the task at hand, shares some conceptual similarities with the task set, and hence interferes with the processing of the relevant dimension (e.g., the color word in the Stroop task). It is often assumed that, in order to give the appropriate response, the processing of the irrelevant dimension must be overcome (Kornblum, Hasbroucq, & Osman, 1990). Ridderinkhof (2002a) proposed that signs of such suppression become manifest in the reaction time (RT) distributions typically obtained in the Simon task. In the most common version of this task, participants must give a right- or left-hand response as a function of the color of a visual stimulus (relevant attribute) presented on either the right or the left of a fixation point (irrelevant attribute). When the positions of the stimulus and the response correspond (compatible trials), RTs are faster and error rates lower, relative to the situation in which these positions do not correspond (incompatible trials). Detailed analysis of RT distributions, however, reveals that the “interference” effect decreases as RTs lengthen: It is large when responses are fast, but decreases as responses get slower (de Jong, Liang, & Lauber, 1994; Ridderinkhof, 2002a; see Fig. 2c in the Results). This effect is best visualized with the delta-plot technique, which plots effect size as a function of response speed: Such delta functions in the Simon task are typically nonlinear, showing an initial increase of the interference effect (positive-going delta plots), followed by a later reversal and negative-going delta plots for the rightmost part of the distribution (see, e.g., Ridderinkhof, 2002b; Wascher, Schatz, Kuder, & Verleger, 2001; Wylie et al., 2010). From a statistical point of view, this nonlinear relationship reflects larger RT variability for the compatible situation, which is associated with the smallest mean, violating the very robust positive linear relationship between mean and standard deviation that is usually observed (Wagenmakers & Brown, 2007; Wagenmakers, Grasman, & Molenaar, 2005; see Schwarz & Miller, 2012, for an evaluation of the different architectures able to account for such a pattern).

One functional interpretation of the decreased interference for longer RTs is that it reflects an active suppression of the activation triggered by the irrelevant attribute (Burle, Possamaï, Vidal, Bonnet, & Hasbroucq, 2002; Ridderinkhof, 2002a). The slower responses benefit from this suppression, whereas fast ones would be left unaffected, explaining the progressive diminution of the interference effect (see van den Wildenberg et al., 2010, for an overview). This explanation has attracted interest, and delta plots have been used as a tool to investigate executive control across experimental conditions or between populations (Wylie et al., 2012; Wylie et al., 2010; see van den Wildenberg et al., 2010, for an overview).

Capitalizing on the suppression idea, Burle et al. (2002) reasoned that the amount of suppression should be greater when incorrect activation is large but the correct response is nonetheless given. They sorted trials as a function of the amount of incorrect response activation. To this end, they recorded the electromyographic (EMG) activity of the muscles involved in response execution, so as to reveal covert incorrect processing (e.g., Burle, Allain, Vidal, & Hasbroucq, 2005; Burle, Roger, Allain, Vidal, & Hasbroucq, 2008; Coles, Gratton, Bashore, Eriksen, & Donchin, 1985). On a substantial number of trials, although the correct response was given, subthreshold EMG activity in the muscles involved in the incorrect response could be observed (see Fig. 1 for an example). Such subthreshold EMG activities (named “partial errors”) reflect an activation of the incorrect response that has been corrected before reaching the response threshold, presumably requiring the involvement of online inhibitory control. In agreement with the view that late, negative-going delta plots reflect active suppression of the irrelevant stimulus dimension, the delta plots calculated for partial errors were even more negative-going, and rather linear. Once partial errors were removed, the delta plots were almost flat (although still slightly decreasing; see Fig. 2f in the Results).

**Fig. 1:**
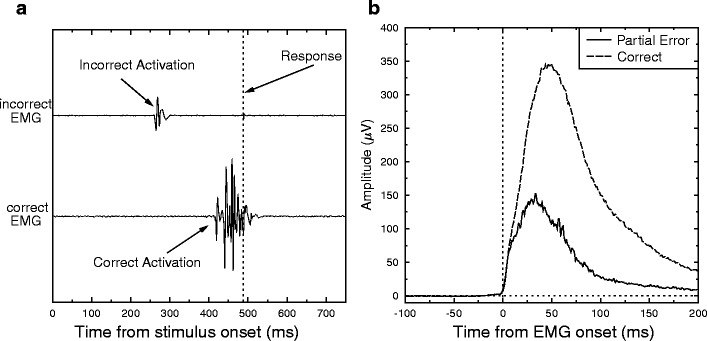
(**a**) Example of a partial-error trial. This graph presents the electromyographic (EMG) activity as a function of time poststimulus, in the muscles involved in the execution of the incorrect (top) and correct (bottom) responses. Time 0 corresponds to stimulus presentation. The vertical black dashed line indicates the mechanical response. Although the correct response was given, one can clearly see EMG activity in the muscle indicating activation of the incorrect response. This activation is, however, too low to trigger an overt error. (**b**) Grand averages of the rectified EMG activity for partial errors (solid line) and correct responses to pure-correct trials (dashed line)

**Fig. 2:**
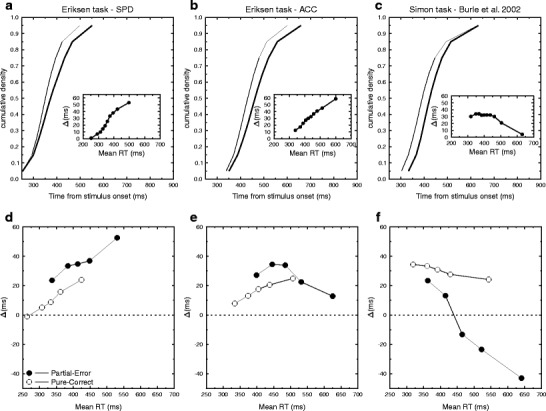
Distributions analysis for the present data and comparison with Simon data. (**a**–**c**) Cumulative density functions and associated delta plots (insets) for the whole distributions of correct RTs, for the SPD and ACC conditions and a Simon task, respectively. The thin lines indicate compatible trials, and the thick lines are incompatible trials. (**d**–**f**) Delta plots for the same data, separately for pure-correct (open circles) and partial-error (filled circles) trials

The link between negative-going delta plots and suppression has been disputed, however, since the need to suppress the irrelevant dimension is supposed to be present in (almost) all conflict tasks, but late negative-going delta plots seem limited to a specific version of the Simon task (Pratte, Rouder, Morey, & Feng, 2010; Ridderinkhof, Scheres, Oosterlaan, & Sergeant, 2005; Wiegand & Wascher, 2005; see Proctor, Miles, & Baroni, 2011, for an overview). We therefore wanted to investigate whether (1) the negative-going nature of Simon activation reflects active inhibition of spatial information in particular, which is not necessarily present in other conflict tasks, or (2) a similar mechanism is present but hidden in the Eriksen task. If Explanation 2 is true, signs of negative-going delta plots should also be observed in the Eriksen task, provided that appropriate conditions are met.

Since partial errors play a critical role in determining the appearance of the negative-going delta plot for the longest RTs, we conjectured that even in the Eriksen task, such a pattern might become visible on trials containing a partial error. We thus studied the RT distributions separately for trials containing or not containing partial errors in a standard Eriksen flanker task.

To better explore the link between negative-going delta plots and executive control, we further manipulated the speed–accuracy trade-off, which is known to affect executive control (Gehring, Goss, Coles, Meyer, & Donchin, 1993; Wylie, Ridderinkhof, Eckerle, & Manning, 2007). Therefore, if the pattern of late, negative-going delta plots relates to executive control, one should expect less-negative-going delta plots in the speed condition.

## Method

### Participants

A group of 16 participants (five women, 11 men, 18–50 years of age) participated in this experiment. They all had a normal (or corrected-to-normal) vision and gave their informed consent.

### Apparatus

The participants were seated in a dark room, facing a panel made of five digit presentation devices (Model No. LTS-3401LP LITE ON) composed of seven-segment light-emitting diodes (to ensure submillisecond accuracy) on which the response signal (the letter H or S ) was presented. The whole display subtended 1.4º of visual angle. The response was a right or a left thumb keypress. The EMG activity of the flexor pollicis brevis of both hands was recorded with two electrodes glued 2 cm apart on the thenar eminences. This activity was amplified, filtered (low/high frequencies cut off at 10 Hz/1 kHz), and digitized online (A/D rate of 2 kHz). The EMG signal was continuously monitored by the experimenter in order to avoid as much as possible any background activity that could hinder small activations during the reaction period. If the signal became noisy, the experimenter immediately asked the participant to relax his or her muscles.

### Procedure

The central digit presentation device (the target) conveyed the response signal (H or S). The four other devices, flanking the target, were distractors. They could be a replication of the target (HHHHH or SSSSS; compatible trials ) or a replication of the alternative response signal (HHSHH or SSHSS; incompatible trials). The four types of stimuli were equiprobable, and the first-order sequential effects for the trial-to-trial transitions were balanced.

All of the participants ran in two experimental sessions that comprised ten blocks of 64 trials. On each session, the participants were asked either to respond very accurately (with the cost of RT lengthening; “ACC” instruction) or to respond very quickly (with the cost of more errors; “SPD” instruction).

The mappings between the target letter and the button were counterbalanced across participants; in each subgroup, half of the participants received the ACC instruction during the first session, whereas the other half received the SPD instruction during the first session.

### Classification of trials

EMG processing has been detailed elsewhere (Burle et al., 2002; Burle et al., 2008; Hasbroucq, Possamaï, Bonnet, & Vidal, 1999), and will only be briefly described here. To detect the smallest incorrect muscular activations, the EMG traces were inspected visually and the EMG onsets were hand-scored. Indeed, although this method is more time consuming, human pattern recognition is superior to automated algorithms (Staude, Flachenecker, Daumer, & Wolf, 2001; van Boxtel, Geraats, van den Berg-Lessen, & Brunia, 1993). It should be emphasized that the experimenter was unaware of the type of trial that he was looking at. Correct trials were sorted into three categories, depending on whether or not EMG activity occurred in the wrong muscle and, when such activity did occur, whether it preceded or followed the correct activity. These categories were labeled “pure-correct” (single EMG activity), “partial errors” (dual-activation trials, with the incorrect activation preceding the correct one by at least 10 ms; see Fig. 1), and “other” trials (Burle et al., 2002; Smid, Mulder, & Mulder, 1990). “Other” trials (about 8%) were discarded from the analysis.

### Distribution analysis

The RT distributions for the pure-correct and partial-error trials were first Vincentized (Jiang, Rouder, & Speckman, 2004; Ratcliff, 1979; Vincent, 1912): The distribution was binned in five classes containing 20% of the trials each, and the mean of each class was computed (Ratcliff, 1979). The delta values were computed on the basis of those Vincentized distributions: The difference between incompatible and compatible RTs in each bin was plotted against the mean RTs for compatible and incompatible trials (Speckman, Rouder, Morey, & Pratte, 2008). One difficulty was to quantify the shape of the resulting delta plots. Previous reported have fitted a linear regression to the whole delta plot (de Jong et al., 1994; Pratte et al., 2010). This, however, does not allow one to extract a potential nonlinear component. Others have computed the between-bin slopes (Ridderinkhof, 2002a; Wylie et al., 2010). This, in turn, does not allow one to quantify the global shape of the delta function. To better quantify the shape of the delta plot, we resorted to the orthogonal-polynomial-contrast approach (Grant, 1956), which, for ordered factors, allows one to test whether a dependent variable is linearly or quadratically (or following higher-order polynomial functions) related to an independent one. If a delta plot presents an initial rise followed by a decrease, its quadratic term should be significant. Although some of the assumptions underlying such analysis might be violated (independence of the values across modalities, homogeneity of variances, and equal spacing), this approach was validated by simulations (see the Results section) and provides essential information to quantify the shape of the delta plot.

### Correction ratio

Partial errors also offer the possibility to directly study the efficiency of online control by evaluating the capacity to overcome incorrect response activation. To quantify this control, Burle et al. (2002) introduced the “correction ratio” (CR), defined as

1 $$ CR=\frac{N_{pe}}{N_{pe}+{N}_{er}} $$

where *N*_pe_ reflects the number of partial errors and *N*_er_ the number of overt errors. In other words, the CR reflects the number of corrected incorrect activations divided by the overall number of incorrect activations (corrected or not). We thus computed the CR under both ACC and SPD conditions, to better evaluate the impact of time pressure on online executive control, and hence to study potential covariations between delta plots and online control efficiency.

## Results

Unless explicitly specified otherwise, all statistical analyses were performed by means of repeated measures canonical analyses of variance (ANOVAs), and planned comparisons were then performed. The percentages of overt and partial errors were arcsine-transformed (Winer, 1971) before being submitted to ANOVAs. Before analyzing the main measures of this study, namely CR and delta plots, we will report the effects of the manipulations of chronometric and accuracy indices.

### Reaction time

The analysis revealed longer RTs on incompatible than on compatible trials [*F*(1, 15)  =  67.46, *p*  <  .0001], and under ACC than under SPD instructions [*F*(1, 15)  =  38.27, *p*  <  .0001]. These factors interacted in an overadditive way—that is, the compatibility effect was larger in the ACC condition [*F*(1, 15)  =  5.81, *p*  <  .03; see Table 1]. The compatibility effects were nonetheless significant for both instructions [*F*(1, 15)  =  57.91, *p*  <  .0001, and *F*(1, 15)  =  53.87, *p*  <  .0001, for ACC and SPD, respectively].

**Table 1:** Effects of the manipulated factors on chronometric and accuracy indices

	RT (ms)		Overt Error (%)		Partial Error (%)	
	ACC	SPD	ACC	SPD	ACC	SPD
Compatible	441	358	1.0	5.7	10.6	19.5
Incompatible	474	382	3.7	14.2	22.8	21.2

### Incorrect response activations

#### Overt errors

The pattern of results was essentially similar to that obtained for RTs (Table 1). The analysis revealed an effect of compatibility [*F*(1, 15)  =  58.48, *p*  <  .0001] and an effect of instruction [*F*(1, 15)  =  421.89, *p* < .0001], and those two factors interacted significantly [*F*(1, 15)  =  23.19, *p*  <  .001], revealing that the increase in the number of errors associated with the incompatible situation was higher for SPD than for ACC instructions. The effects of compatibility were nonetheless significant for both types of instructions [*F*(1, 15)  =  18.46, *p*  <  .001, and *F*(1, 15)  =  88.77, *p* < .0001, for the ACC and SPD instructions, respectively].

#### Partial errors

Besides overt errors, we also investigated the impact of the manipulated factors on the probability of occurrence of partial errors (Table 1). This analysis revealed a main effect of compatibility [*F*(1, 15)  =  59.5, *p*  <  .0001], a main effect of instructions [*F*(1, 15)  =  16.85, *p*  <  .001], and an interaction between these two factors [*F*(1, 15)  =  11.10, *p*  <  .005]. However, contrary to the result observed for overt errors, the interaction showed that the increase in the number of partial errors due to compatibility was lower in the SPD than in the ACC condition. Contrast analyses revealed an effect of instructions in the compatible condition [SPD  >  ACC; *F*(1, 15)  =  51.8, *p*  <  .0001], but no effect in the incompatible condition [*F*(1, 15)  =  2.12, *p*  =  .17].

### Impact of time pressure

In order to obtain reliable CR and delta-plot analyses, one needs a minimum number of partial errors. Four participants among the 16 had fewer than ten partial errors in at least one condition, and were hence discarded. The analysis was thus conducted on 12 participants (the results of the preceding analyses for those 12 participants were the same as those for the 16 participants, except that the interaction between compatibility and instructions that became significant, *F*(1, 11)  =  7.5, *p*  =  .019.

#### Distribution analysis

These results are presented in Fig. 2a and b (Fig. 2c presents the data obtained in the Simon task by Burle et al., 2002, for the sake of comparison). The analysis on pure-correct trials revealed an effect of bins [*F*(4, 44)  =  10.82, *p*  <  .001], no effect of instruction [*F*(1, 11)  =  1.5, *p*  =  .25], and no interaction (*F*  <  1). To better characterize the shape of the delta plot, trend analyses were performed using an orthogonal polynomial approach (Grant, 1956). The linear components were significant for both the ACC [*F*(1, 11)  =  7.5, *p*  <  .02; Fig. 2e] and the SPD [*F*(1, 11)  =  10.6, *p*  <  .01; Fig. 2d] instructions. The quadratic components were far from significance (all *F*s  <  1). The patterns of results were largely different for partial-error trials: No main effect of instruction was observed [*F*(1, 11)  =  2.22, *p*  =  .16] and no effect of bin (*F*  <  1). Importantly, these two factors interacted significantly [*F*(4, 44)  =  4.26, *p*  <  .01]. Trend analysis revealed that the quadratic component was significant for the ACC condition [*F*(1, 11)  =  5.49, *p*  <  .04; Fig. 2e], indicating that after an initial increase, the delta values decreased. The linear component was far from the significance level (*F*  <  1). For the SPD condition, the linear component was highly significant [*F*(1, 11)  =  17.7, *p*  <  .002; Fig. 2d], but not the quadratic one (*F*  <  1).^1^

#### Correction ratio

**Fig. 3:**
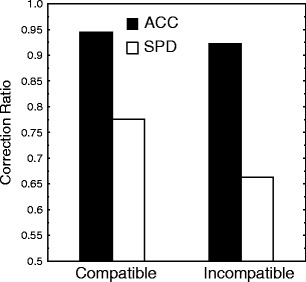
Correction ratios for compatible and incompatible trials, separated for the ACC (filled bars) and SPD (empty bars) conditions

The overall CR indicates that about 93% of incorrect response activations were ultimately corrected in the ACC condition, and about 71% in the SPD condition (Fig. 3). These two values differed significantly [*F*(1, 11)  =  53.23, *p*  <  .001]. Compatibility also affected the CR [*F*(1, 11)  =  10.38, *p*  <  .01] and interacted with condition [*F*(1, 11)  =  10.66, *p*  <  .01]. Contrast analysis revealed that the CRs differed between compatible and incompatible trials only in the SPD condition [*F*(1, 11)  =  16.46, *p*  <  .002, and *F*(1, 11)  =  1.03, *p*  =  .33, for SPD and ACC, respectively]. The absence of a compatibility effect in the ACC condition replicates in the Eriksen task data already obtained in the Simon task (Burle et al., 2002).

## Discussion

RT distribution analysis in the Simon task has revealed that the interference effect decreases as RTs lengthen. Although this pattern was interpreted as reflecting an active suppression of the irrelevant dimension of the stimulus (Ridderinkhof, 2002a, 2002b), this view has been disputed, since the need for suppression is supposed to be present in (almost) all conflict tasks, but late negative-going delta plots seemed limited to a specific version of the Simon task (Pratte et al., 2010; Wiegand & Wascher, 2005; see Proctor et al., 2011, for an overview).

Here we have shown, however, that although no negative-going delta plots are observable on the whole-trial distribution in the Eriksen flanker task (Fig. 2), such a pattern appears when one looks specifically at trials containing a partial error, extending to the Eriksen task what has already been reported in the Simon task (Burle et al., 2002). First, this indicates that late, negative-going delta plots are not restricted to a specific version of the Simon task, but generalize to other tasks (see also Roelofs, Piai, & Rodriguez, 2011), although with different strengths. Second, those results also emphasize the critical role played by partial-error trials in such a statistical pattern. Indeed, in both the Simon (Burle et al., 2002) and Eriksen (present data) tasks, negative-going delta plots are mainly seen on those trials.

If similar mechanisms are at play in both tasks, why are their contribution to the whole RT distributions on the two tasks different? A first possibility could be a mere proportion effect, with more partial errors being associated with more-negative-going delta plots. This simple view is, however, unlikely, since the proportions of partial errors obtained in the present report were very similar to the ones obtained in the Simon task (around 15%; see van den Wildenberg et al., 2010, for an overview).

The difference appears to be more deeply rooted in some aspects of the tasks themselves. Indeed, in the Simon task (Fig. 2c and f), delta-plot slopes are largely negative-going for the whole RT range of partial-error trials, and the interference effect even reverses for long RTs. On pure-correct trials, although the negative-going pattern is clearly reduced when compared to the overall RT (see Fig. 2f), the delta slopes are still slightly negative-going. In the Eriksen task, although late negative-going delta plots are observed for partial errors, the strength of the effect is clearly an order of magnitude smaller than for Simon task. Moreover, for pure-correct trials, no negative-going delta plots are observed (see Fig. 2d and e). It thus appears that the mechanism leading to the interference decrease, whatever its nature, is present but weaker on the Eriksen task.

What is the link, if any, between late negative-going delta plots and response suppression? From a factual point of view, late negative-going delta plots are mainly due to partial errors, in which response suppression is likely present. Furthermore, the presence of late negative-going delta plots covaries with an independent measure of control such as the CR, since a reduction of the CR in the SPD condition is associated with the disappearance of the late negative-going delta plots. Thus, the present data confirm the usefulness of EMG recording in better quantifying online control, but they also suggest that, although late negative-going delta plots are associated with control, this link is rather indirect. A further dissociation comes from the SPD condition. Indeed, even under speed pressure, the incorrect response had to be overcome in the case of partial errors. If late negative-going delta plots reflect incorrect-response suppression, it should have also occurred in this case. This might, however, come from the different natures of partial errors between ACC and SPD conditions. Indeed, the numbers of partial errors did not differ between compatible and incompatible trials in the SPD condition, suggesting that, under this condition, incorrect activation is not stimulus driven, but may rather reflect guesses from the participants (Gratton, Coles, & Donchin, 1992). Although this deserves more investigation, this may point toward different correction sensitivities for guesses and stimulus-driven incorrect activations.

What could be the nature of the mechanism producing negative-going delta plots? Insights might be found in recent modeling of Eriksen tasks. Two recent models (Hübner, Steinhauser, & Lehle, 2010; White, Ratcliff, & Starns, 2011), both formulated within the diffusion framework, have implemented variable drift-diffusion models of the Eriksen task, in which the rate of evidence accumulation changes during the course of a trial. One of these models (White et al., 2011) clearly implements a suppression of the irrelevant dimension (the flankers), but at the perceptual level. Since the initial accumulation drifts toward the incorrect response on incompatible trials, one may be able to identify an equivalent of partial errors in those models (see Burle et al., 2008, for a similar logic) and to assess whether negative-going delta plots could also be predicted by such models on “partial-error” trials. Such questions, currently under investigations, are arguably beyond the scope of the present study, however.

## Footnotes

1 One reviewer correctly pointed out that, given the small amount of data included, and since some of the assumptions were not fulfilled in this type of data, one may wonder about the robustness of the trend effect. We thus ran 10,000 simulations, with the very same number of data per participant, using Weibull distributions, known to theoretically produce positively increasing delta plots. We then checked how many time a negative-bended quadratic term was significant. This occurred in about 0.1% to 0.2% of the cases. Hence, the significant quadratic component cannot be considered a result of random fluctuations.
